# Snakebite envenoming: A systematic review and meta-analysis of global morbidity and mortality

**DOI:** 10.1371/journal.pntd.0012080

**Published:** 2024-04-04

**Authors:** Afsana Afroz, Bodrun Naher Siddiquea, Hasina Akhter Chowdhury, Timothy NW Jackson, Andrew D. Watt

**Affiliations:** 1 Australian Venom Research Unit, Department of Biochemistry and Pharmacology, Faculty of Medicine, Dentistry and Health Sciences, University of Melbourne, Melbourne, Australia; 2 Department of Epidemiology and Preventive Medicine, School of Public Health and Preventive Medicine, Faculty of Medicine, Nursing and Health Sciences, Monash University, Clayton, Australia; College of Health Sciences, Bayero University Kano, NIGERIA

## Abstract

**Background:**

Snakebite envenoming represents a significant and often neglected public health challenge, particularly in rural communities across tropical and subtropical regions. An estimated 1.2–5.5 million people are envenomed by snakebites annually. More than 125,000 of these bites are fatal, and 3–4 times as many results in disability/disfigurement. Despite its prevalence, collecting accurate epidemiological data on snakebite is challenging. This systematic review and meta-analysis collates global epidemiology data on snakebite morbidity and mortality.

**Methods:**

Medline, Embase, Cochrane and CINAHL Plus databases were searched for articles published between 2001–2022. Pooled incidence and mortality were obtained using random effects modelling, heterogeneity (I^2^) was tested, and sensitivity analyses performed. Newcastle-Ottawa Scale assessed study quality.

**Results:**

Out of the four databases, 5,312 articles were found. After removing duplicates, 3,953 articles were screened by title and abstract and 65 articles containing information on snakebite epidemiology, encompassing 663,460 snakebites, were selected for analysis. The people most at risk for snakebite were men (59%), engaged in agricultural labour (27.5%), and residing in rural areas (66.7%). More than half (57%) of the reported bites resulted in envenoming. Incidents occurred frequently in the summer season (38.5%), during daytime (56.7%), and bites were most often to the lower limb (56.4%). Envenoming severity was frequently mild (46.7%), treated in hospital (68.3%), and was treated with anti-venom (64.7%). The pooled global incidence and mortality was 69.4 /100,000 population (95%CI: 36.8 to 101.9) and 0.33/100,000 population (95%CI, 0.14 to 0.52) per year, respectively.

Stratified by continents, Asia had the highest incidence of 130.7/100,000 population (95%CI: 48.3 to 213.1) while Europe has the lowest with 0.7/100,000 population (95%CI: -0.2 to 1.5). The highest mortality was reported in Asia at 0.96/100,000 population (95% CI: 0.22 to 1.70), and Africa 0.44/100,000 population (95%CI: -0.03 to 0.84). Incidence was highest among inhabitants of lower-middle-income countries 132.7/100,000 population (95%CI: 55.4 to 209.9) while mortality was highest in low-income countries at 0.85/100,000 population (95% CI: -0.06 to 2.31).

**Conclusion:**

Incidence and mortality rates noted here highlight the global impact of snakebite and underscore the critical need to address the burden of snakebite envenoming. It also reveals that while reported snakebite incidence was higher in lower-middle-income countries, the burden of mortality was greatest among inhabitants of low-income countries, again emphasising the need for greater efforts to tackle this neglected tropical disease.

## Author summary

Snakebite envenoming poses a significant public health challenge, especially in rural tropical and subtropical areas. Annually, an estimated 1.2–5.5 million people are envenomed, with over 125,000 fatalities and many more suffering disabilities. Collecting accurate epidemiological data on snakebite is challenging. This systematic review and meta-analysis compile global data on snakebite morbidity and mortality.

After screening thousands of articles, 65 were selected, covering 663,460 snakebites. Men in agricultural areas were most at risk, with rural dwellers bearing the brunt. Most bites occurred in summer, during the day, and affected the lower limb. Envenoming severity was typically mild, often treated in hospitals with anti-venom.

Globally, the pooled incidence was 69.4/100,000 population per year and mortality was 0.33/100,000 population per year. Asia reported the highest incidence and mortality rates. Incidence was highest in lower-middle-income countries, while mortality was greatest in low-income countries.

These findings underscore the urgent need to address the burden of snakebite envenoming, particularly in resource-limited settings. Efforts must focus on prevention, treatment, and strengthening healthcare systems to mitigate the impact of this neglected tropical disease.

## Introduction

Snakebite envenoming affects millions of people worldwide and is a significant source of mortality [1], primarily in rural and agricultural communities of tropical and subtropical countries [1]. In 2019, the World Health Organization (WHO) set a target to halve the number of deaths and cases of snakebite envenoming by 2030 [2]. According to the WHO, there are approximately 5.4 million snakebites and 1.8–2.7 million cases of envenomation globally each year, including 81,410–137,880 deaths and around three times as many individuals suffering from permanent disfigurement and/or disabilities, including limb amputations [1]. Regions such as Sub-Saharan Africa, Southeast Asia, and South Asia experience the highest incidence of snakebite, with up to 200,000 cases of envenoming estimated in Asia and 435,000 to 580,000 cases estimated to occur across Africa annually [1]. In South Asia, India experiences the highest mortality rate attributable to snakebite envenomation with approximately 45,900 deaths reported each year [3].

Several factors contribute to the risk of snakebite, including occupations that result in frequent exposure to snakes, such as agricultural work, farming, and herding, living in rural areas with close proximity to snake habitats, and insufficient knowledge about snakebite prevention. The lack of identification of venomous snakes and appropriate first aid measures, initial management by traditional healers, delay in reaching hospital and limited access to healthcare, including antivenoms, in rural areas further exacerbate the consequences of snakebite envenoming [4,5].

Despite the scale of snakebite across the world, reliable incidence and mortality data remain largely unavailable across snakebite endemic areas across rural equatorial regions; reliable data are instead mostly limited to a few developed countries where bites are relatively rare. Having information on the number of snakebites, envenomings, deaths, and long-term morbidity, is crucial for evaluating the impact of snakebite in these areas and for developing management guidelines, planning healthcare resources (especially antivenom availability), and providing appropriate training to healthcare professionals for effective snakebite treatment [6]. Furthermore, if we are to achieve the strategic goals set by the WHO to reduce snakebite-related deaths and disabilities by 50% by the year 2030 [1], comprehensive data on the incidence of snakebite and mortality rates are urgently needed. Therefore, this systematic review and metanalysis aims to assess and summarise the incidence of snakebite and resultant mortality available from published data globally.

### Methodology

**Fig 1:**
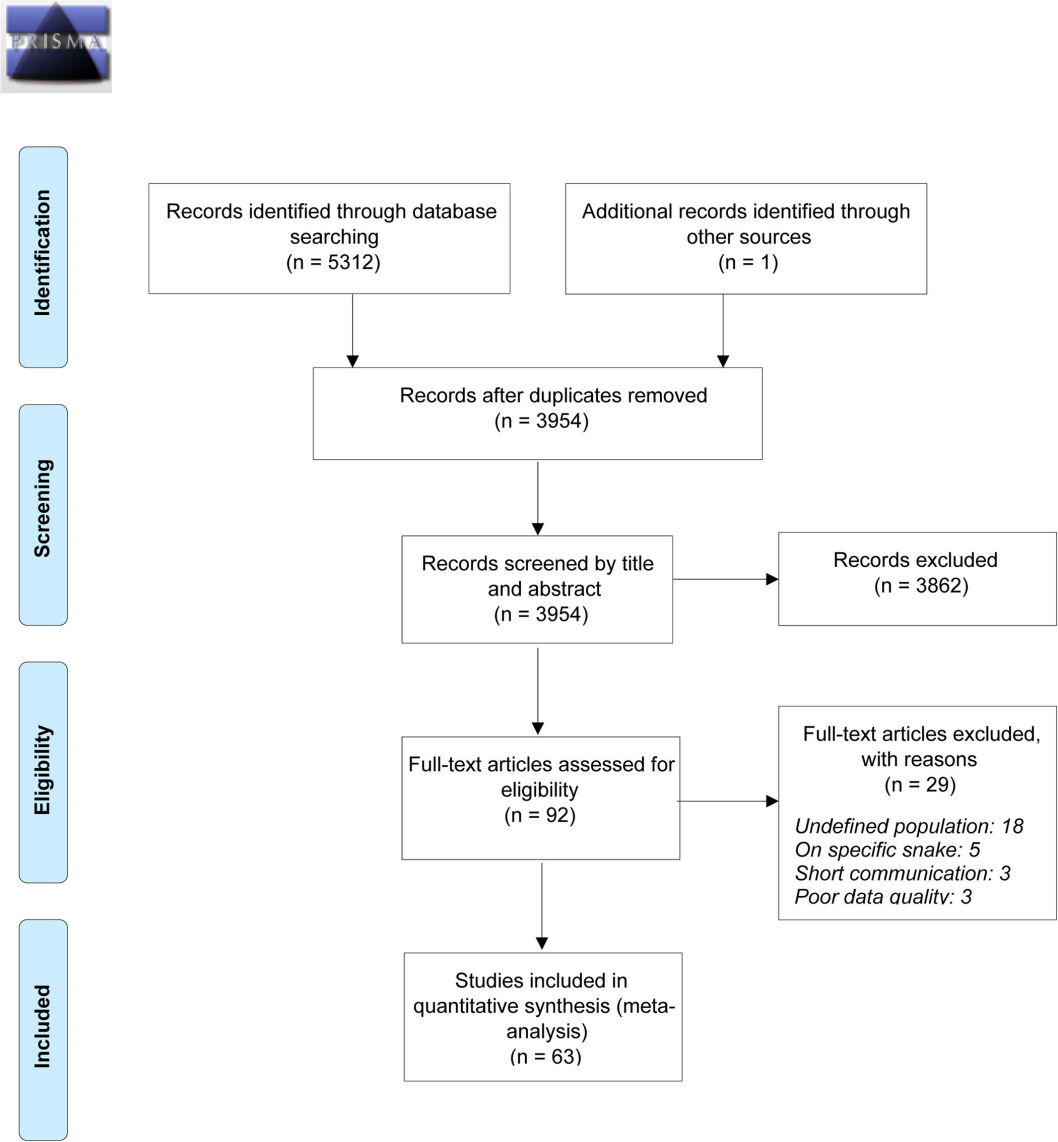
PRISMA 2009 Flow Diagram.

This systematic review and meta-analysis adhered to the guidelines for Preferred Reporting Items for Systematic Reviews and Meta-Analyses (PRISMA) [7] and was registered with the International Prospective Register for Systematic Review (PROSPERO ID: CRD42022377613). The PRISMA flow diagram illustrates the identification and screening process of studies for inclusion in the meta-analysis (***Fig 1***). Two reviewers (BS and HAC) independently assessed all articles, extracted data, and completed the PRISMA checklist, which is included as supporting information (*S1 PRISMA Checklist*). As this review is based on published literature, no ethics approval was necessary.

### Search strategy and study selection

Two authors (BNS and HAC) independently conducted a search for articles published between 01^st^ January 2001 and 31^st^ December 2022 on Medline, Embase, CINAHL Plus, and Cochrane. The authors used key terms recommended by the senior librarian at the University of Melbourne to conduct the search. The key search terms were the following: [Incidence OR prevalence OR epidemiology OR risk factors] AND [Snakebite OR Snake venom]. Searched articles were stored and managed using citation software EndNote X20. A detailed description of the search strategy has been provided as supplementary information (*S1 Search strategy*). The articles retrieved from the search were screened for eligibility based on their titles and abstracts by both BNS and HAC independently. Any articles that did not meet the criteria (outlined below) were excluded. Additionally, relevant publications’ reference lists were manually reviewed to identify any other studies. Inclusion and exclusion criteria were clearly defined for the study selection process. After a full read, BNS, HAC, and AA discussed and selected the final set of 65 articles. In case of any disagreement, the study lead, AA, made the final decision.

### Selection criteria

To be eligible for inclusion, studies had to meet the following criteria: 1. Study of any design with published data, 2. Contained estimations of snakebite incidence and/or mortality due to snakebite envenoming in terms of actual number, incidence per 100,000 population or incidence per 100,000 population per year.

Studies were excluded if they met the following criteria: 1. Overlapping articles or duplicate data, editorials, reviews, short communication, case reports, preprints. 2. Meta-analyses or review articles. 3. Studies with undefined population or outcome. 4. Studies lacking sufficient methodological precision, 5. Focused on specific venomous snake or clinical complications.

For included studies, data containing name of first author, publication year, geographic location, study design and study period, all demographic details, number of snakebite cases and deaths due to snakebite were identified. Data were extracted and transferred to Microsoft Excel by two authors (BNS and HAC) for each eligible study.

### Outcome measure

The primary outcome was the incidence of snakebite. All reported number were retrieved from the articles. Where the absolute annual number of snakebite envenoming cases reported by a given study was available, the incidence rate per 100,000 population was calculated using the country population or the catchment area population for the reporting year by referring to online sources.

The secondary outcome was mortality due to snakebites. Information on mortality was gathered by analysing the articles. Of the articles that reported total number of deaths were considered and Mortality rates (per 100,000 population) were calculated using the country populations of the reporting year as the denominator.

### Risk of bias and quality assessment in individual studies

The terms risk of bias and quality assessment are commonly used interchangeably. Researchers use various tools to evaluate safeguards for internal validity and explore the potential reliability of evidence generated within a study [8,9]. In this review, using the Newcastle-Ottawa Scale (NOS) tool, BNS and HAC evaluated the risk of bias and quality for each eligible study [10]. ***The NOS is based on an eight-item score divided into three domains and is currently the most frequently utilised tool to assess study quality and risk of bias*. *It also offers flexibility for modification according to specific subjects* [8]**. This tool utilises a "star system" that assesses a study based on three main perspectives: the selection of study groups, the comparability of those groups, and the ascertainment of exposure of interest (outcome) for cohort, observational or cross-sectional studies. ***The overall quality of the study is rated as “good*,*” “fair*,*” or “poor*,*” based on the reviewing authors’ judgments about the study quality and risk of bias item for each included study***; details can be found as supporting information (*S1 Quality assessment*). Minor discrepancies were resolved by lead author, AA.

### Data analysis

The lead author (AA) analysed the data extracted from each study using Stata V.17 (StataCorp., College Station, Texas, United States of America). The senior author (AW) cross-checked the analysis, and any discrepancies were resolved through discussion. The overall incidence and mortality estimates, along with corresponding 95% confidence intervals, were calculated using random-effects models of restricted maximum-likelihood method [11] (i.e., an open model in which effects are not constant). In the presence of heterogeneity (as expected and observed), random-effect models have superior properties and are more conservative than fixed-effect models [12]. The fixed-effect model assumes that differences in observed effects result from sampling error, whereas the random-effects model suggests that the true effect might vary among studies due to inherent differences (heterogeneity) among studies. This approach makes it possible to estimate the variables by accounting for the heterogeneity of results and the weight of each study according to the number and type of population under review [13]. The χ2-test on Cochran’s Q statistic was used to test for between-study heterogeneity. The H and I^2^ indices were used to calculate this statistic, with I^2^ representing the percentage of total heterogeneity across studies based on true between-study differences rather than on chance. Conventionally, I^2^ values of 0–25% indicate low heterogeneity, 26–75% indicate moderate heterogeneity, and 76–100% indicate substantial heterogeneity [14]. To identify the possible sources of substantial/considerable heterogeneity, subgroup analysis was carried out for the following covariates: continent, economic classification [15], study design (Observational, Cross sectional and Cohort) and study setting (Community based, Database and Hospital based).

A sensitivity analysis was conducted using the ‘leave-one-out’ method to ascertain the influence of any single study on the overall result [16]. Publication bias was assessed by visual inspection of Begg’s funnel plots, Begg’s and Egger’s regression test [17,18]. The publication bias was declared in situations where the p-values from both Begg’s and Egger’s regression test were significant. All p-value <0.5 was considered as statistical significance.

## Results

A comprehensive search of databases including Medline (2246), Embase (2837), CINAHL Plus (229), and Cochrane (0) added a total of 5,312 published articles from 2001 to 2022. After removing duplicates (1359), the remaining 3,954 articles were screened based on titles and abstracts and 3862 were excluded, as represented in **Fig 1**. From the remaining 92 articles, a thorough evaluation was conducted on a selection of articles to determine their eligibility for inclusion. As a result, 26 articles were excluded, leaving a final set of 63 [5,19–80] studies from 29 countries for this study. **Table 1** provides a description of the included studies.

**Table 1:** Descriptions of the included studied.

Study ID	Country	Study period (years)	Study design	Study population	Snake bite cases	Outcome included (I, M)	First author	Publication year
1	Brazil	9	Cohort	8843000	1063	I, M	Albuquerque PL et al [19]	2013
2	Cameroon	1	Cross sectional	9924	66	I, M	Alcoba G et al [20]	2020
3	Nepal	1	Cross sectional	63454	166	I, M	Alcoba G et al [21]	2022
4	Morocco	16	Cohort	28550000	1423	I, M	Arfaoui et al [22]	2009
5	India	5	Cohort	27300000	6555	I, M	Bhargava S et al [23]	2018
6	South Africa	2.5	Observational	300000	333	I, M	Blaylock R [24]	2003
7	USA	5	Cohort	4440000	674	I, M	Buchanan et al [25]	2021
8	Ghana	5	Cohort	2457792	2973	I	Ceesay B et al [26]	2021
9	Brazil	11	Cohort	2449024	5568	I, M	Ceron K et al [27]	2021
10	Turkey	10	Cohort	63240194	550	I	Cesaretli et al [28]	2010
11	Morocco	5	Cohort	32223000	873	I, M	Chafiq F et al [29]	2016
12	Taiwan	5	Cohort	22952400	4647	I, M	Chen CK et al [30]	2015
13	Brazil	12	Cohort	189512052	326481	I, M	Chippaux JP [31]	2015
14	Brazil	10	Cohort	3168027	3909	I, M	Costa M et al [32]	2019
15	Bosnia and Herzegovina	23	Cohort	4,384,662	341	I, M	Curic I et al [33]	2009
16	Australia	8.6	Cohort	140000	216	I	Currie BJ et al [34]	2004
17	Iran	7	Cohort	293996	50	I, M	Dehghani et al [35]	2012
18	Iran	1	Observational	75373855	5172	I, M	Dehghani R et al [36]	2014
19	Iran	10	Cohort	79960000	53787	I, M	Dehghani R et al [37]	2014
20	Iran	1	Cohort	79960000	4917	I, M	Dehghani R et al [37]	2014
21	Iran	5	Cohort	66000	195	I	Ebrahimi V et al [38]	2018
22	Sri Lanka	1	Cross sectional	165665	677	I	Ediriweera et al [39]	2020
23	Mozambique	20	Cohort	7544	297	I, M	Farooq et al [40]	2022
24	Kenya	1	Observational	3613429	176	I, M	Francis Okumu Ochola et al [41]	2018
25	India	1	Cross sectional	402095	145	I, M	Gajbhiye R et al [42]	2019
26	Burkina Faso	5	Cohort	17051002	114126	I, M	Gampini S et al [43]	2016
27	Ecuador	10	Cohort	13000000	14720	I, M	Gonzalez-Andrade F and Chippaux JP [44]	2010
28	India	5	Cohort	580320	497	I, M	Gupt A et al [45]	2015
29	Sudan	5	Observational	39446096	63160	I, M	H. Khalid and R. S. Azrag et al [46]	2021
30	Nicaragua	5	Cohort	5900000	3286	I, M	Hansson et al [47]	2010
31	Morocco	15	Cohort	30896566	2053	I, M	Hattimy et al [48]	2018
32	Bangladesh	1	Cross sectional	819429	90	I, M	Hossain J et al [49]	2016
33	Bulgaria	9	Cohort	622867	68	I, M	Iliev YT et al [50]	2014
34	Pakistan	4	Observational	1136044	695	I, M	Jamali et al [51]	2022
35	Sweden	10	Cohort	25630000	1548	I, M	Johnston CI et al [52]	2017
36	Nepal	4	Cohort	5560000	265	I	Karki et al [53]	2019
37	Iran	5	Observational	133099	102	I	Kassiri H et al [54]	2019
38	India	1	Observational	263426	245	I, M	Kharat R and Kedare R [55]	2020
39	Brazil	4	Cohort	185235	351	I, M	Leite Rde S et al [56]	2013
40	Croatia	21	Cohort	496395	542	I, M	Lucsic B et al [57]	2006
41	Brazil	6	Cohort	15772000	2431	I, M	Machado C et al [58]	2012
42	Nepal	3	Observational	2356820	6993	I, M	Magar CT et al [59]	2013
43	Myanmar	1	Cross sectional	19877	24	I	Mahmood MA et al [60]	2018
44	India	2	Cross sectional	1952546	4871	I, M	Majumder et al [61]	2014
45	Brazil	6	Cohort	213159	304	I	Oliveira HFA et al [62]	2013
46	Brazil	1	Observational	407319	118	I	Oliveira LP et al [63]	2020
47	Nepal	1	Observational	249735	274	I, M	Pandey DP [64]	2018
48	Nepal	0.67	Observational	2500000	476	I, M	Pandey et al [65]	2022
49	Nepal	1	Cross sectional	1372	32	I	Parajuli et al [66]	2022
50	Ecuador	5	Cohort	103697	133	I	Patino RSP et al [67]	2022
51	Panama	2	Cohort	236489	390	I	Pecchio M et al [68]	2018
52	Bangladesh	1	Cross sectional	18857	98	I, M	Rahman R et al [69]	2010
53	India	8	Cohort	610577	409	I	Rai A et al [70]	2021
54	Brazil	2	Cohort	1562409	92	I	Roriz et al [71]	2017
55	USA	3	Observational	287201314	450	I	Ruha AM et al [5]	2017
56	India	5	Cohort	2145572	1633	I, M	Sarkhel S et al [72]	2017
57	Costa Rica	2	Observational	4764064	475	I, M	Sasa M and Segura Cano SE [73]	2020
58	Brazil	1	Observational	190755799	28716	I, M	Schneider et al [74]	2021
59	South Korea	6	Cohort	50326620	1335	I, M	Senek MZF et al [75]	2019
60	Brazil	1	Observational	137722	133	I, M	Silva et al [76]	2019
61	Brazil	8	Cohort	3168027	3019	I, M	Tavares AV et al [77]	2017
62	Cameroon	1	Observational	1409348	516	I, M	Tchoffo et al [78]	2019
63	Lao PDR (Laos)	1	Cross sectional	9856	35	I	Vongphoumy I et al [79]	2015
64	Lao PDR (Laos)	1	Cross sectional	7150	79	I	Vongphoumy I et al [79]	2015
65	South Africa	5	Cohort	3000000	879	I	Wood et al [80]	2016

From the selected 63 [5,19–80] articles two of the studies had consecutive information that was treated as separate studies and thus we have reported 65 studies in the overall analysis. As detailed in **Table 2**, a total of 663,460 snakebite cases were identified. Among these cases, approximately 58.9% were reported as male based on information from 57/65 (87.69%) studies. The age range of the affected individuals spanned from 0 to 92 years, as reported by 23/65 (35.38%) studies. Additionally, based on data from 19/65 (29.23%) and 27/65 (41.54%) studies, respectively, individuals in farming or agriculture professions (27.5%) and those living in rural areas (66.7%) were identified as being more vulnerable to snakebite.

**Table 2:** Summary of the included studies by demography, seasonal and clinical features.

Variables	Categories	N	Number of studies reported	n	%
**Total**		**663460**	**65**		
Gender		167274	57		
	Male			98477	58.9
	Female			57118	34.1
	Unknown			11679	7.0
Age		23019	23		
Occupation		12617	19		
	Farming/agriculture			3466	27.5
	Laboure			545	4.3
	Housewife			650	5.2
	Service			314	2.5
	Unemployed			151	1.2
	Student			1042	8.3
	Others			1575	12.5
	Unknown			4874	38.6
Area of residence		27016	27		
	Rural			18020	66.7
	Peri-urban			6976	25.8
	Urban			508	1.9
	Unknown			1512	5.6
Snake type		49920	34		
	Venomous			28607	57.3
	Non-venomous			10310	20.7
	Unknown			11003	22.0
Location/place of bites		9185	12		
	Agriculture field			1574	17.1
	Road or path			1215	13.2
	Home			2027	22.1
	Outdoor working area			152	1.7
	Fishing			1138	12.4
	Others			1648	17.9
	Unknown			1431	15.6
Season		13457	18		
	Summer			5175	38.5
	Monsoon			2852	21.2
	Spring			2253	16.7
	Autumn			1053	7.8
	Winter			490	3.6
	Unknown			1634	12.1
Bite time		7380	15		
	Day			4181	56.7
	Night			2568	34.8
	Unknown			631	8.6
Location of bite		54254	39		
	Lower limb			30575	56.4
	Upper limb			12940	23.9
	Others			3261	6.0
	Unknown			7478	13.8
Severity of envenomation		42864	27		
	Mild			20000	46.7
	Moderate			12100	28.2
	Severe			4232	9.9
	No envenomation			205	0.5
	Unknown			6327	14.8
First-aid received		11148	10		
	Yes			3158	28.3
	No			7974	71.5
	Unknown			16	0.1
Treatment type		3328	13		
	Traditional			759	22.81
	Formal treatment (in hospital)			2272	68.3
	No treatment			280	8.4
	Others			17	0.5
Use of anti-venom		54483	33		
	Yes			35266	64.7
	No			9923	18.2
	Unknown			9294	17.1

Of the 49,920 cases that were examined across 34/65 (52.31%) studies, 57.3% of individuals were bitten by a venomous snake. According to 12/65 (18.46%) studies, 22.1% of these cases occurred at home, while 17.1% occurred in fields, and 13.2% occurred on roads or paths.

In terms of seasonal trends, 38.5% of snakebites were reported during summer, followed by 21.2% during monsoon season and 16.7% during spring, as reported by 18/65 (27.69%) studies. Additionally, as stated by 15/65 (23.08%) and 39/65 (60.00%) studies respectively, over half of all snakebites (56.7%) occurred during the daytime and 56.4% affected the lower limbs. In terms of severity, approximately 46.7% of cases as reported in 27/65 (41.54%) studies were considered mild, while 28.2% were classified as moderate and 9.9% were reported as severe.

Considering treatment details, 10/65 (15.38%) studies reported on first-aid treatment, 13/65 (20.00%) studies covered the type of treatment, and 33/65 (50.77%) studies reported on use of antivenom. Of the reported studies, more than two-thirds (71.5%) of the cases did not receive first-aid, 68.3% were treated in a hospital setting, and 64.7% received antivenom. Supplementary information containing detailed study-specific findings can be accessed for further insights (*S1 Study-wise description*).

### Meta-analysis of incidence and mortality

**Fig 2:**
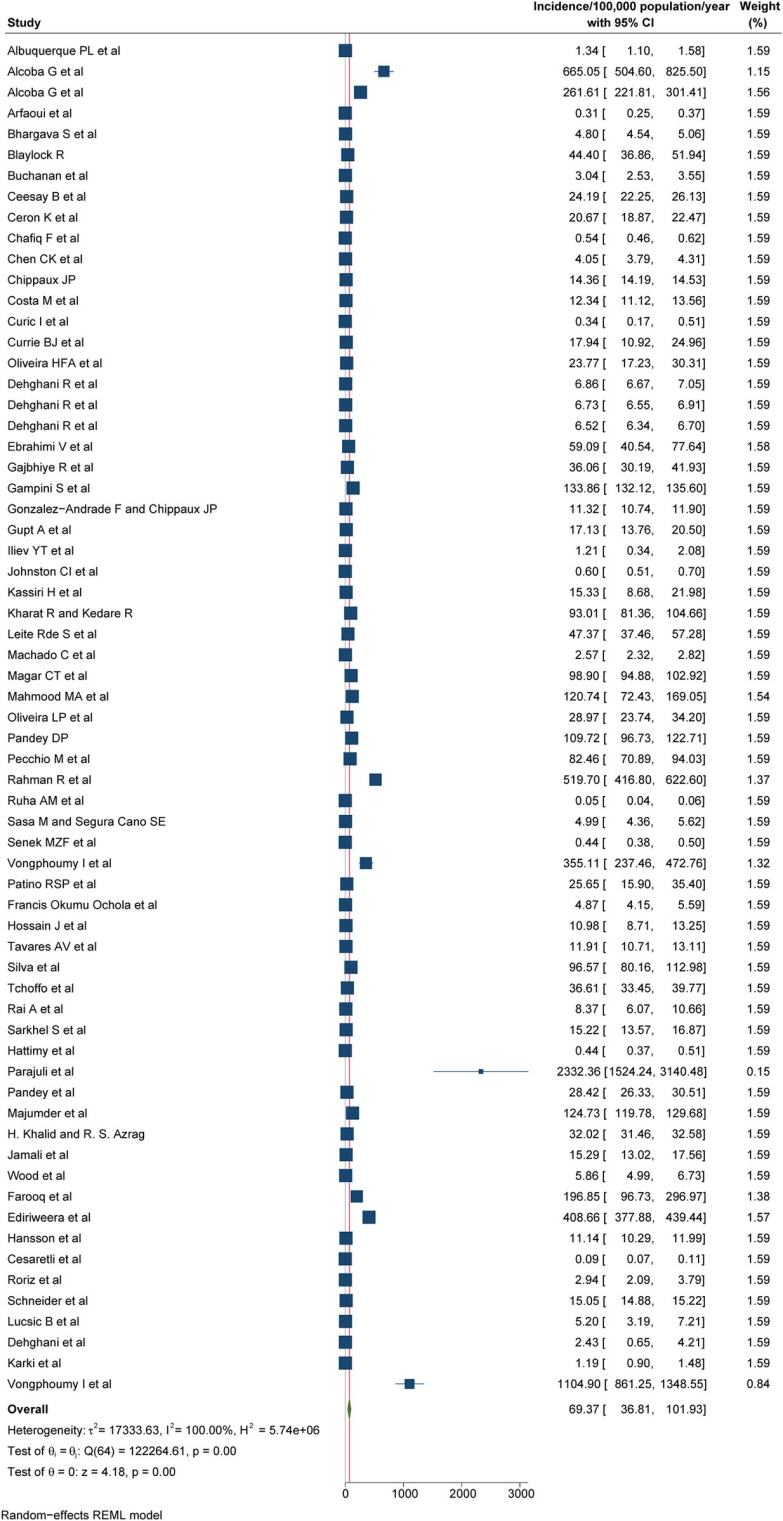
Pooled incidence of snakebite, REML-Restricted Maximum Likelihood.

**Fig 3:**
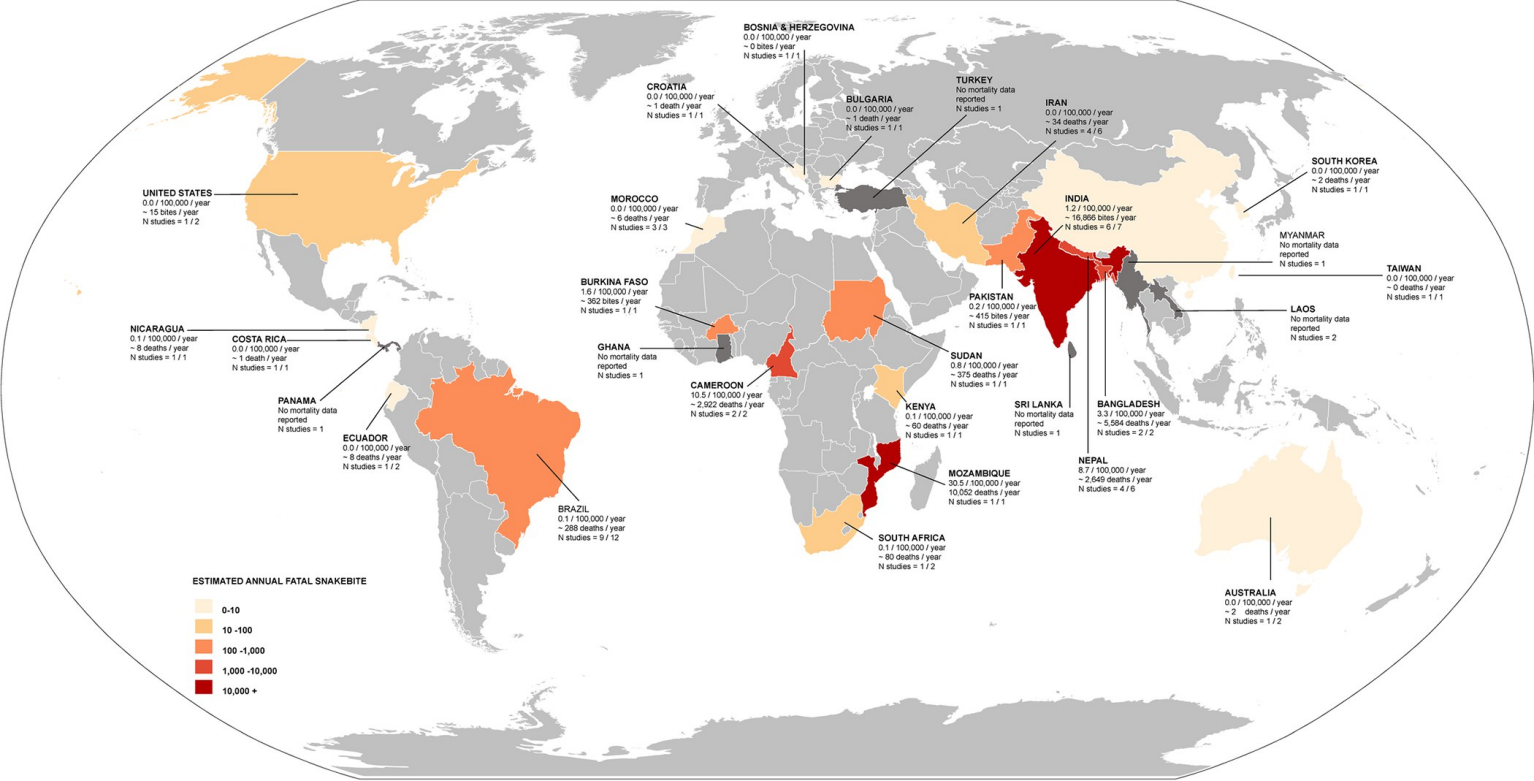
World map with incidence of snakebites per 100,000 population per year across the globe. Study number pre county have been provided. For countries with multiple studies, average incidence per 100,000 population per year have been provided and noted. (The direct link to the base layer of the map: https://commons.wikimedia.org/wiki/File:BlankMap-World.svg).

According to our most conservative estimates from 65 studies, the pooled global incidence of snakebite was 69.4/100,000 population (95% CI: 36.8 to 101.9; **Fig 2**), also mapped on **Fig 3**. Stratified by continents (**Table 3**), Asia has the highest incidence of 130.7/100,000 population (95% CI: 48.3 to 213.1), followed by Africa 84.2/100,000 population (95% CI: -6.0 to 174.5), South America 21.7/100,000 population (95% CI: 9.8 to 33.7), North America 19.9/100,000 population (95% CI: -10.2 to 50.1), Oceania 7.1/100,000 population (95% CI: -2.3 to 17.1) and Europe 0.7/100,000 population (95% CI: -0.2 to 1.5). The incidence was highest among inhabitants of lower-middle income countries at 132.6/100,000 population (95% CI: 55.4 to 209.9), followed by low income countries 72.5/100,000 population (95% CI: -47.8 to 192.8), middle countries 22.4/100,000 population (95% CI: 8.4 to 36.5), upper-middle income countries 15.8/100,000 population (95% CI: 2.5 to 29.2) and the lowest in high-income countries 12.4/100,000 population (95% CI: -4.5 to 29.2; **Table 3**). The pooled incidence of the studies that scored good, fair, or poor in our quality assessment was 183.7/100,000 population (95% CI: 19.9 to 347.5), 76.3/100,000 population (95% CI: 13.2 to 139.5), and 24.3/100,000 population (95% CI: 12.3 to 36.3), respectively (**Table 3**). High heterogeneity was observed to the reported incidences of snakebites (I^2^ >75%), with the absence of publication bias considering both Beggs test and Egger’s regression test (p<0.05). None of the following stratification helped identify the studies primarily responsible for the high heterogeneity: geographical location, economical classification, study quality, study setting and study design.

**Table 3:** Sub-group analysis for the overall pooled incidence of the included studies.

Variable	No of studies	Incidence/100,000 population/ year (95% CI*), p-value	I^2^	Egger test (p-value)	Beggs test (p-value)
**Continent**					
Africa	12	84.2 (-6.0 to 174.5), <0.01	100.0	0.001	0.4507
Asia	29	130.7 (48.3 to 213.1), <0.01	100.0	0.001	0.1486
Europe	2	0.7 (-0.2 to 1.5), 0.05	73.25	-	
North America	5	19.9 (-10.2 to 50.1), <0.01	99.99	0.026	0.2207
Oceania	3	7.1 (-2.3 to 17.1), <0.01	98.61	0.001	0.2963
South America	14	21.7 (9.8 to 33.7), <0.01	99.99	0.001	0.2284
**Economy**					
Low-income countries	2	72.5 (-47.8 to 192.8), <0.01	99.99		
Lower-middle income countries	34	132.6 (55.4 to 209.9), <0.01	100.0	0.001	0.4767
Middle-income countries	12	22.4 (8.4 to 36.5), <0.01	99.99	0.001	0.3037
Upper-middle countries	8	15.8 (2.5 to 29.2), <0.01	99.99	0.001	0.9015
High-income countries	9	12.4 (-4.5 to 29.2), <0.01	100.0	0.001	0.9170
**Study Quality**					
Good	12	183.7 (19.9 to 347.5), <0.01	100.0	0.001	0.9453
Fair	21	76.3 (13.2 to 139.5), <0.01	100.0	0.001	0.2639
Poor	32	24.3 (12.3 to 36.3), <0.01	100.0	0.001	0.8840
**Study setting**					
Registry/database	14	306.2 (79.0 to 533.4), 0.01	100.0	0.001	0.7426
Community based	26	42.4 (7.8 to 76.9), 0.01	100.0	0.001	0.3780
Hospital based	25	24.4 (13.3 to 35.1), 0.01	100.0	0.001	0.6913
**Study design**					
Observational	16	35.4 (17.7 to 53.1), <0.01	100.0	0.001	0.7526
Cross sectional	12	422.5 (147.2 to 697.9), <0.01	100.0	0.001	0.3727
Cohort	37	18.9 (9.3 to 28.5), 0.01	100.0	0.001	1.0000

**Fig 4:**
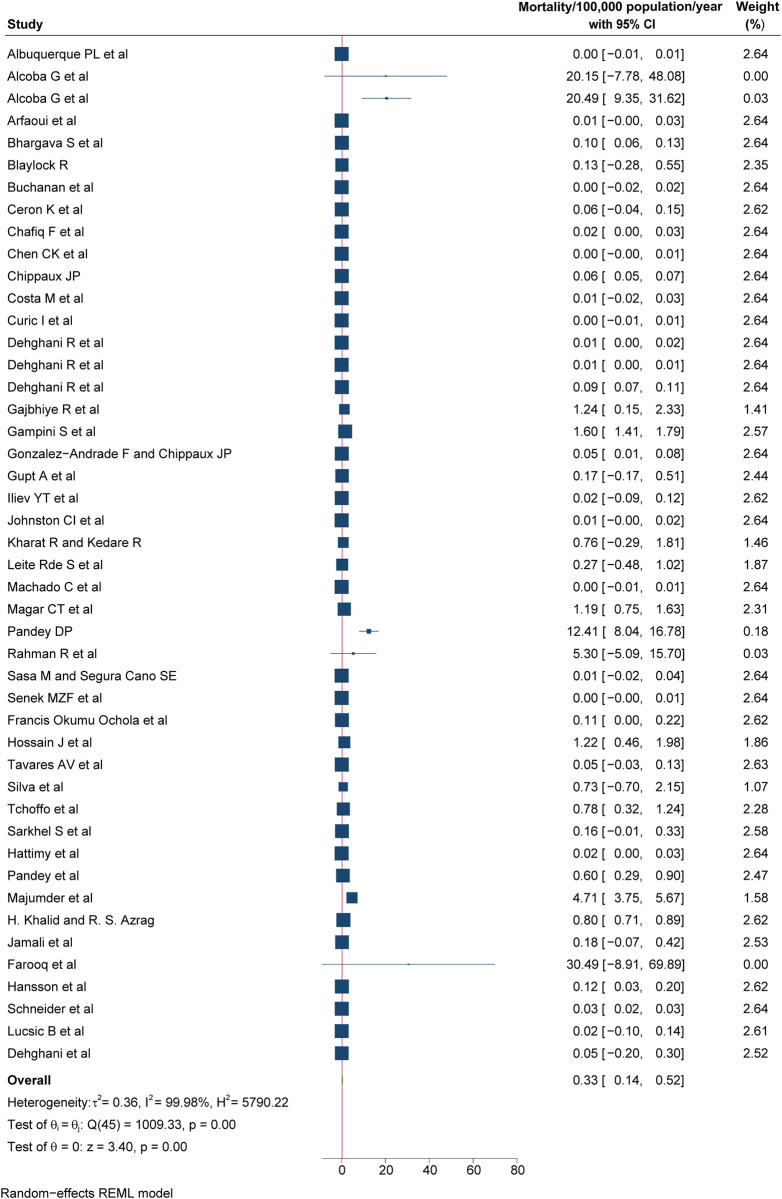
Pooled mortality of snakebite, REML-Restricted Maximum Likelihood.

**Fig 5:**
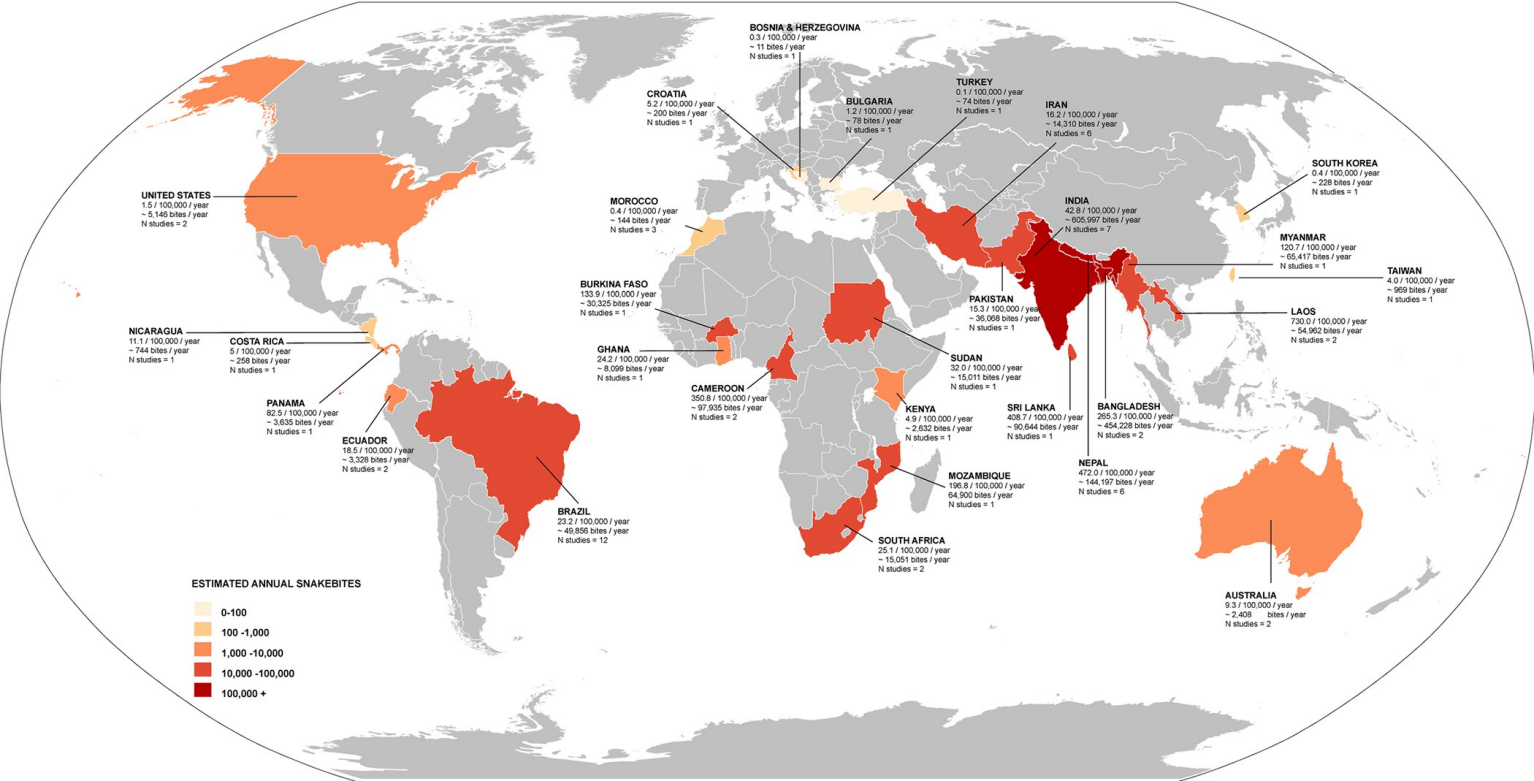
World map with mortality of snakebites per 100,000 population per year across the globe. Study number pre county have been provided. For countries with multiple studies, average mortality per 100,000 population per year have been provided and noted. (The direct link to the base layer of the map: https://commons.wikimedia.org/wiki/File:BlankMap-World.svg).

Based on the 46/65 (69.23%) studies that provided mortality data, the overall global mortality due to snakebites was estimated at 0.33/100,000 population (95% CI: 0.14 to 0.52; **Fig 4**) and mapped on world map (**Fig 5**). When stratified by continents (**Table 4**), Asia had the highest mortality at 0.96/100,000 population (95% CI: 0.22 to 1.7), followed by Africa at 0.44/100,000 population (95% CI: -0.03 to 0.84), North America at 0.03/100,000 population (95% CI: -0.02 to 0.08) and South America at 0.03/100,000 (95% CI: 0.01 to 0.05) had a similar mortality rate, whereas Europe and Oceania had the similar at 0.01/100,000 population (95% CI: -0.01 to 0.02) and 0.01/100,000 population (95% CI: -0.00 to 0.02), respectively. Among different income categories (**Table 4**), the mortality was highest among inhabitants of low-income countries at 0.85/100,000 population (95% CI: -0.60 to 2.31), followed by lower-middle income countries at 0.74/100,000 population (95% CI: 0.25 to 1.23), middle-income countries at 0.02/100,000 population (95% CI: 0.01 to 0.04), upper-middle income countries at 0.01/100,000 population (95% CI: -0.01 to 0.04), and the lowest rate was observed in high-income countries at 0.00/100,000 population (95% CI: -0.00 to 0.01). The pooled mortality rate was lowest for studies scored as poor with 0.03/100,000 population (95% CI: 0.01 to 0.05), followed by studies scored as fair 0.44/100,000 population (95% CI: 0.17 to 0.70), and was 1.21/100,000 population (95%CI: —0.49 to 2.92) for studies scored as good in the quality assessment (**Table 4**). The heterogeneity of the mortality studies was high (I^2^ >75%), and did not reduce by geographical location, economical classification, study quality, study setting and study design stratification, with mostly the absence of publication bias considering both Beggs test and Egger’s regression test (p<0.05).

**Table 4:** Sub-group analysis for the overall pooled mortality of the included studies.

Variable	No of studies	Mortality/100,000 population/ year (95% CI*), p-value	I^2^	Egger test (p-value)	Beggs test (p-value)
**Continent**					
Africa	10	0.44 (-0.03 to 0.84), <0.01	99.93	0.027	0.2105
Asia	19	0.96 (0.22 to 1.70), <0.01	100.0	<0.001	0.0501
Europe	2	0.01 (-0.01 to 0.02), 0.91	0.00		
North America	3	0.03 (-0.02 to 0.08)	84.44	0.017	0.2963
Oceania	2	0.01 (-0.00 to 0.02), 0.87	0.01	1.000	
South America	10	0.03 (0.01 to 0.05), <0.01	89.58	0.149	0.8580
**Economy**					
Low-income countries	2	0.85 (-0.60 to 2.31), <0.01	99.48	1.000	0.0059
Lower-middle income countries	25	0.74 (0.25 to 1.23), <0.01	99.99	<0.001	0.0500
Middle-income countries	9	0.02 (0.01 to 0.04), <0.01	90.87	0.179	0.6022
Upper-middle countries	4	0.01 (-0.01 to 0.04), <0.01	50.45	0.015	0.2207
High-income countries	6	0.00 (-0.00 to 0.01), <0.01	0.08	0.484	0.4524
**Study Quality**					
Good	7	1.21 (-0.49 to 2.92), <0.01	99.99	<0.001	0.0715
Fair	17	0.44 (0.17 to 0.70), <0.01	99.97	0.004	0.5366
Poor	22	0.03 (0.01 to 0.05), <0.01	94.26	**<0.001**	**0.0068**
**Study setting**					
Registry/database	9	2.61 (-0.01 to 5.32), <0.01	100.0	0.0007	0.7545
Community based	20	0.23 (0.02 to 0.44), <0.01	99.97	**0.0001**	**0.0125**
Hospital based	17	0.06 (0.02 to 0.09), <0.01	95.45	0.0001	0.1494
**Study design**					
Observational	12	0.28 (0.07 to 0.50), <0.01	99.92	0.0001	0.2437
Cross sectional	7	3.38 (0.16 to 6.60), <0.01	98.31	0.0029	1.0000
Cohort	27	0.15 (0.02 to 0.29), <0.01	99.94	**0.0001**	**0.0059**

### Sensitivity analysis

**Fig 6:**
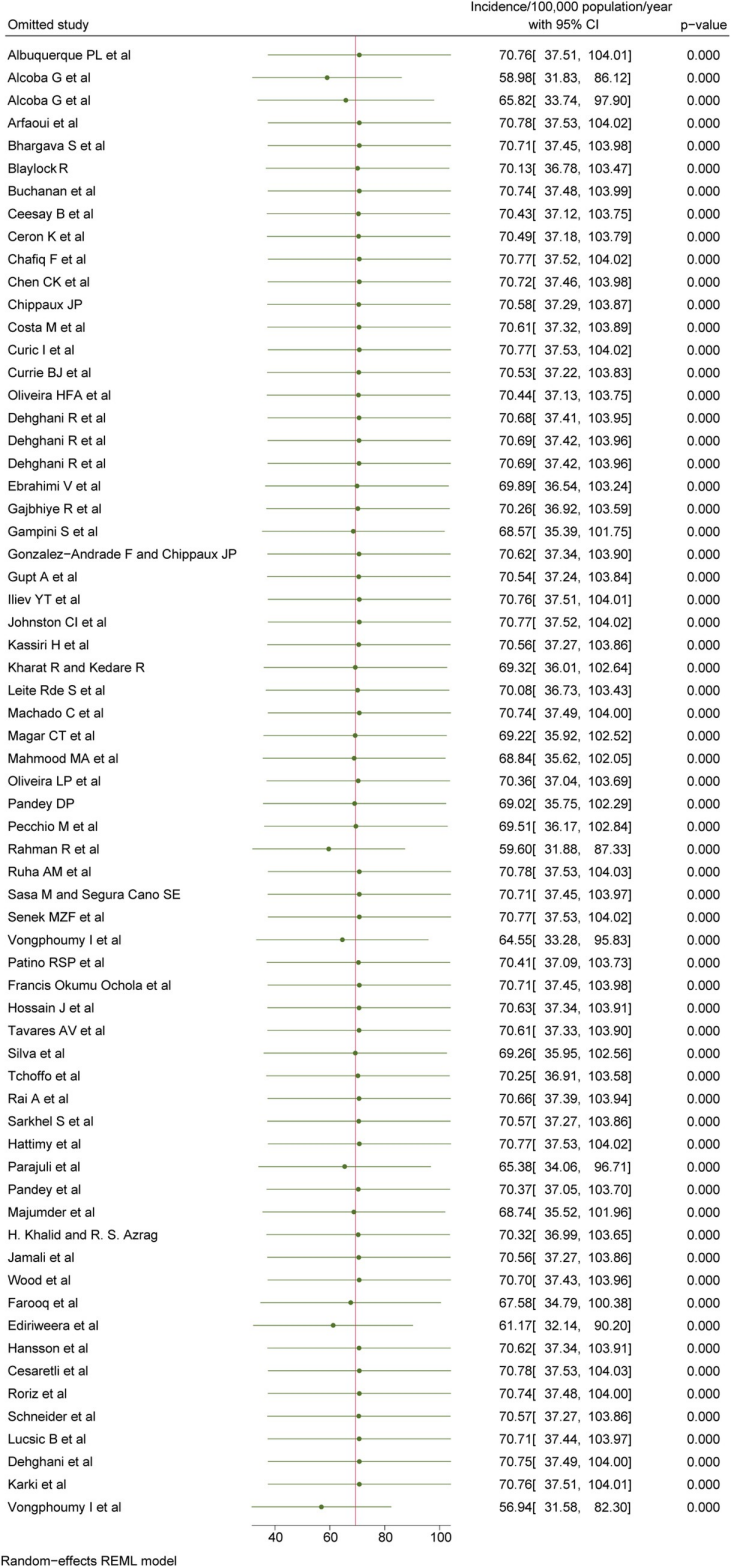
Sensitivity analysis for the studies reporting incidence.

Sensitivity analysis indicated that the pooled incidence estimation was relatively robust to the exclusion of any one study from the overall meta-analysis and did not change by more than 10% (**Fig 6**) except when leaving out the following four individual studies: Alcoba G et al [pooled incidence: 58.9 (95% CI: 31.8 to 86.1)] [20], Rahman R et al [pooled incidence: 59.6 (95% CI: 31.9 to 87.3)] [69], Ediriweera et al [pooled incidence: 61.2 (95% CI: 32.3 to 103.9)] [39] and Vongphoumy et al [pooled incidence: 56.9 (95% CI: 31.6 to 82.3)] [79]. The overall heterogeneity was unaffected (I^2^ = 100.0%).

**Fig 7:**
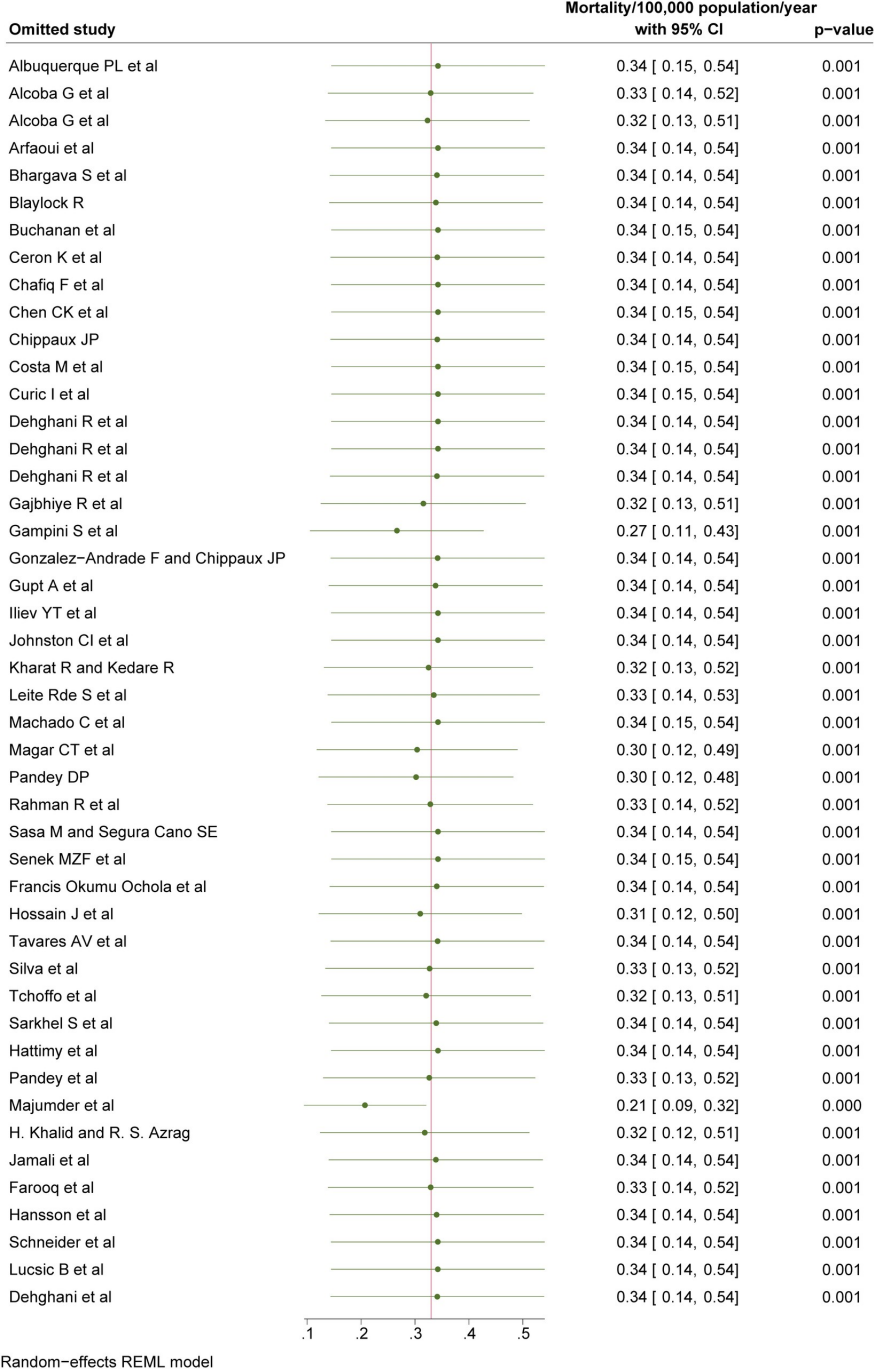
Sensitivity analysis for the studies reporting mortality.

Sensitivity analysis for the mortality status showed that the pooled mortality did not significantly change after excluding studies one by one, and the change was within 10% with the exception of two studies: Gampini et al. [pooled mortality: 0.27 (95% CI: 0.11 to 0.43)] [43], and Majumder et al. [pooled mortality: 0.21 (95% CI: 0.09 to 0.32)] [61] (**Fig 7**). However, the overall heterogeneity was unaffected with the removal of these two studies (I^2^ = 100.0%). This indicates insensitivity of the overall pooled-Incidence rate.

## Discussion

Snakebites occur when humans and snakes come into contact as a consequence of their intersecting behavioural ecologies. As both human and snake ecologies differ seasonally and geographically, the number of interactions and their outcomes vary widely over the course of the year and across the planet. This review focused on the morbidity and mortality of snakebites based on available published data globally. We estimate the global incidence of snakebite as 69.4/100,000 population (95% CI: 36.8 to 101.9), and mortality as 0.33/100,000 population (95% CI: 0.14 to 0.52). The highest estimated morbidity rates of snakebite were observed in Asia and Africa, with the lowest incidence rates observed in Europe. Similarly, Asia had the highest mortality rate at 0.96/100,000 population (95% CI: 0.22 to 1.7), followed by Africa at 0.44/100,000 population (95% CI: -0.03 to 0.84). These findings were in line with those of Kasturiratne et al., who estimated the highest number of deaths due to snakebite to occur in South Asia, followed by sub-Saharan Africa, while the lowest numbers of deaths were estimated for Australasia, Southern Latin America, and Western Europe [6].

A study using regional data estimates, which were derived from country-specific data within a defined region, published global estimates of the incidence of venomous snakebite indicating that between the years 1990 and 2019, there were approximately 1,200,000 to 5,500,000 snakebite envenoming incidents, resulting in 63,400 deaths, worldwide. Our study examined the period from 2000 to 2022, focusing on all snakebite (venomous and non-venomous). During this period, the estimated number of snakebite incidents was 18,390,000 and mortality was 1,390,000. In this review, among the 49,920 cases from 34 studies, it was found that 57.3% of individuals were envenomed. Previous studies have suggested that snakebites resulting in envenoming ranged from 12% to 87% of the total number of snakebites [6].

Certain human activities and geographical locations significantly increase the risk of encountering snakes. Individuals residing in tropical regions and engaged in rural lifestyles and agricultural professions are at a higher risk of snakebite. In the 19th century, the bite incidence was very high among farmers [81], but agricultural mechanisation has undoubtedly reduced the risk of snakebites significantly. Especially in Europe, where agricultural activities are no longer a common association of snakebite [82]. This is reinforced by the results of this review, in which we found that 22.1% of cases occurred at home, while 17.1% occurred in agriculture fields. This has somewhat altered the circumstances surrounding snakebites, especially among populations susceptible to bites, particularly children. Seasonal patterns reveal a broader distribution of snakebite incidents during spring and summer [83]. This systematic review also found that 39.5% of the total number of snakebites occurred during summer, followed by 21.2% during the monsoon season and 16.7% during spring. It should be noted that while monsoon seasons occur in tropical regions, they do not occur in more temperate regions where snakebites are also endemic. Thus, the seasonality of snakebite should be considered on a regional basis.

According to research conducted by Ralph et al. in 2019, the mortality rate following a venomous snakebite increases if antivenom is not administered within six hours [84]. This review identified Asia as the region with the highest recorded mortality rates, followed by Africa. Many countries in South Asia are classified as lower-middle income countries and a combination of ecological factors, socioeconomic vulnerability, and limited capacity within their healthcare systems is likely to contribute to the burden of snakebite envenoming within these regions. For example, individuals may turn to traditional healers or visit clinics with inadequate knowledge on how to treat snakebite envenoming or which lack the necessary antivenom for life-saving treatment [84,85,86]. Countries in Sub-Saharan Africa have been reported to face similar challenges. The production of antivenom may also be insufficient for the incidence of snakebite in a region or may be disproportionately available in private clinics rendering them unaffordable to those most at risk of being envenomed. The presence of political conflict and humanitarian crises further exacerbate the situation [87,88]. In contrast, South America, and Europe exhibit lower mortality rates from snakebites. In Europe, where high-quality healthcare services are readily accessible and well-distributed, individuals are more inclined to seek prompt medical attention. Snake bite mortality in South America may be lower due to the presence of improved snakebite management systems, which include the development of locally effective antivenoms [89,90]. This proactive approach contributes to better outcomes in managing snakebite incidents. In this review, the pooled incidence and mortality for the hospital-based studies showed the lowest compared to community-based studies and studies with a data source from a database or registry support the community health seeking behaviour where people do not seek conventional medical care and are therefore missed in hospital record.

In terms of public health, gaining a comprehensive understanding of the disease burden is essential for effectively addressing its consequences. Previous evidence has highlighted the significant impact of snakebite envenoming, with an estimated 63,400 deaths (95% UI 38,900–78,600) and 2.9 million years of life lost (YLLs; 1.8 million–3.7 million) in 2019. These statistics establish snakebite envenoming as one of the deadliest neglected tropical diseases (NTDs) based on the Global Burden of Disease study in 2019 [91].

The consequences of snakebite envenoming include the need for antivenom, hospitalization, intensive care unit care, surgery, long-term sequelae (*i*.*e*., disability/disfigurement), and death. To estimate the total number of snakebites, we relied on data regarding the prevalence of snakebite from studies conducted in various regions worldwide. However, the estimated number of bites exhibited considerable variation amongst studies, likely due to methodological differences. This heterogeneity implies that our estimation of the total number of snakebites is only a rough approximation. The actual magnitude of the snakebite burden may not be accurately represented in recorded data, as a significant proportion of individuals with asymptomatic, mild, or even life-threatening bites may not seek medical treatment at hospitals and health clinics that collate data pertaining to these injuries. Furthermore, it is impossible to determine, particularly in community surveys, whether "all bites" encompasses bites from non-venomous snakes and/or dry bites from venomous snakes. While these non-envenoming bites may not contribute significantly to the overall disease burden, the opportunity cost of the bite can still have adverse effects on the victims and their households. Furthermore, data regarding the incidence of non-envenoming snakebite are potentially useful as an index of the prevalence of antagonistic encounters between humans and snakes (of which bites resulting in envenoming are a subset). These data are undoubtedly underreported, but we encourage researchers to collect them, including as much information as possible regarding the circumstances of snakebites, whether or not envenoming results. A deeper understanding of the *ecology* of snakebite may be one pathway towards reducing its prevalence.

The strength of this review is the fact that our analyses encompassed a comprehensive selection of 65 studies, shedding light on the global incidence and mortality of snakebites across all regions. In the study by Kasturiratne et al on the global burden of snakebite, several assumptions were used to ensure the representativeness of the data. In instances where no data were accessible for a specific country to calculate the incidence, the lowest incidence rate within a neighbouring country was used. Additionally, a country was considered as free of snakebites if there was no literature indicating occurrences since 1985. Furthermore, a country was considered to have no mortality due to snakebites, even if reports of snakebites existed, if no mortality statistics had been reported to the WHO mortality database from 1990 to the present date [6]. However, in this review, absolute annual number of snakebite envenoming cases and country population or the catchment area population for the reporting year reported by a given study was used to calculate the incidence rate per 100,000 population, online sources were used when country population or the catchment area population was not available in the study. However, this study retains certain limitations that need to be acknowledged. Firstly, there was a high level of heterogeneity observed in the evaluated outcomes. Incidence data vary widely across countries and among studies, with differences in study methodology contributing most notably to this variability along with geographical location, and the circumstances surrounding the snakebite incidents. It is important to note that most of the articles examined were not standardised epidemiological studies. However, this heterogeneity may serve as an indicator of variations among the studied populations, study design and settings, helping to identify the underlying causes for these differences. Secondly, the results of the sensitivity analysis highlighted the impact of including or excluding the study conducted by Alcoba G et al, Rahman R et al, Ediriweera et al and Vongphoumy et al. The inclusion of these studies led to an overall pooled incidence rate rage of 69.4/100,000 population, while their exclusion resulted in a lower pooled incidence rate in a range between 56.9/100,000 population and 61.2/100,000 population. Therefore, caution is needed when interpreting and generalising the findings. Thirdly, it is important to note that this study only included articles published in the English language. Consequently, there is a possibility that relevant studies published in other languages may have been excluded from analysis. Overall, these strengths and limitations should be considered when interpreting the findings of this review.

## Conclusion

The actual magnitude of snakebite burden may not be accurately represented in recorded data due to methodological differences among included studies. However, incidence and mortality rates mentioned in this report serve as a stark reminder of the worldwide significance of snakebites and emphasise the urgent necessity to address the burden they impose. These findings also shed light on a crucial disparity: although reported snakebite incidence was higher in upper-middle-income countries the highest mortality rates occurred among residents of low-income countries. This striking contrast further emphasizes the imperative for intensified investigation of interventions aimed at combatting this neglected tropical disease.

## Supporting information

S1 PRISMA Checklist PRISMA 2020 Checklist. (DOCX)

S1 Search Strategy Ovid Medline (R). (DOCX)

S1 Quality Assessment Quality assessment results for the Newcastle-Ottawa Scale (NOS) quality assessment tool for observational, cohort and cross-sectional studies. (DOCX)

S1 Study-wise Description Study-wise description of the included studies by demography, seasonal and clinical features. (XLSX)
