# Supplementary material for: Snakebite envenoming: A systematic review and meta-analysis of global morbidity and mortality
Source: PLoS Negl Trop Dis. 2024 Apr 4;18(4):e0012080. doi: 10.1371/journal.pntd.0012080 (PMC11020954; doi:10.1371/journal.pntd.0012080)
Supplement: S1 Search Strategy — (DOCX) [file pntd.0012080.s002.docx]

Search strategy: Ovid Medline (R)

| **#** | **Searches** |
| --- | --- |
| 1 | exp Snake Venoms/ or (snake* adj3 venom*).tw. |
| 2 | Crotalid Venoms/ or Snake Bites/ or Elapid Venoms/ or (((crotalid or elapid) adj3 venom*) or (snake bite* or snakebite*)).tw. |
| 3 | exp Scorpion Venoms/ or (scorpion* adj3 venom*).tw. |
| 4 | exp Spider Venoms/ or ((spider* adj3 venom*) or spider bite*).tw. |
| 5 | exp arthropod venom/ or ((arthropod* or ant or ants or bee or bees or wasp*) adj3 (venom* or sting or stings or stinging)).tw. |
| 6 | exp Cnidarian Venoms/ or exp Marine Toxins/ or exp Marine Toxins/ or ((cnidarian or marine or jellyfish or fish) adj3 (venom* or sting or stings or stinging)).tw. |
| 7 | 1 or 2 or 3 or 4 or 5 or 6 |
| 8 | exp Epidemiology/ or epidemiolog*.tw. |
| 9 | exp Prevalence/ or prevalen*.tw. |
| 10 | exp Incidence/ or incidence.tw. |
| 11 | Risk Factors/ or risk*.tw. |
| 12 | 8 or 9 or 10 or 11 |
| 13 | 7 and 12 |
| 14 | limit 13 to yr="2000 -Current" |
| 15 | limit 14 to "humans only (removes records about animals)" |
