# Supplementary material for: Snakebite envenoming: A systematic review and meta-analysis of global morbidity and mortality
Source: PLoS Negl Trop Dis. 2024 Apr 4;18(4):e0012080. doi: 10.1371/journal.pntd.0012080 (PMC11020954; doi:10.1371/journal.pntd.0012080)
Supplement: S1 Quality Assessment — (DOCX) [file pntd.0012080.s003.docx]

| **Study author** | **Selection** | | | | | | **Comparability** | **Outcome** | | | **Total score (out of 7)** | **Quality** |
| --- | --- | --- | --- | --- | --- | --- | --- | --- | --- | --- | --- | --- |
|  | **Criteria 1** | **Criteria 2** | **Criteria 3** | **Criteria 4** | **Criteria 5** | **Criteria 6** | **Criteria 7** | **Criteria 8** | **Criteria 9** | **Criteria 10** |  |  |
| Blaylock R | 1 | N/A | 1 | 1 | 1 | 1 | 0 | 1 | N/A | N/A | 6 | Good |
| Dehghani R et al | 1 | N/A | 1 | 1 | 0 | 0 | 0 | 1 | N/A | N/A | 4 | Fair |
| Karlson-Stiber C et al | 1 | N/A | 1 | 1 | 0 | 0 | 0 | 1 | N/A | N/A | 4 | Fair |
| Kassiri H et al | 1 | N/A | 1 | 1 | 0 | 0 | 0 | 1 | N/A | N/A | 4 | Fair |
| Kharat R and Kedare R | 0 | N/A | 1 | 1 | 0 | 0 | 0 | 1 | N/A | N/A | 3 | Poor |
| Magar CT et al | 1 | N/A | 1 | 1 | 0 | 0 | 0 | 1 | N/A | N/A | 4 | Fair |
| Oliveira LP et al | 1 | N/A | 1 | 1 | 0 | 0 | 0 | 1 | N/A | N/A | 4 | Fair |
| Pecchio M et al | 1 | N/A | 1 | 1 | 0 | 0 | 0 | 1 | N/A | N/A | 4 | Fair |
| Ruha AM et al | 1 | N/A | 1 | 1 | 1 | 1 | 0 | 1 | N/A | N/A | 6 | Good |
| Sasa M and Segura Cano SE | 1 | N/A | 1 | 1 | 1 | 0 | 0 | 1 | N/A | N/A | 5 | Fair |
| Ochola et al | 1 | N/A | 1 | 1 | 0 | 0 | 0 | 1 | N/A | N/A | 4 | Fair |
| Silva et al | 1 | N/A | 1 | 1 | 0 | 0 | 0 | 1 | N/A | N/A | 4 | Fair |
| Tchoffo et al | 1 | N/A | 1 | 1 | 1 | 0 | 0 | 1 | N/A | N/A | 5 | Fair |
| Pandey et al | 1 | N/A | 1 | 1 | 1 | 1 | 0 | 1 | N/A | N/A | 6 | Good |
| H. Khalid and R. S. Azrag | 1 | N/A | 1 | 1 | 0 | 0 | 0 | 1 | N/A | N/A | 4 | Fair |
| Jamali et al | 1 | N/A | 1 | 1 | 0 | 0 | 0 | 1 | N/A | N/A | 4 | Fair |
| Schneider et al | 1 | N/A | 1 | 1 | 0 | 0 | 0 | 1 | N/A | N/A | 4 | Fair |

S3 (A): Assessment of studies (Observational) using the Newcastle-Ottawa Scale (NOS) tool, Grading: good: 6-7, fair: 4-5, poor: 0-3

| **Study author** | **Selection** | | | | **Total selection** | **Comparability** | **Outcome** | | | **Total outcome** | **Quality** |
| --- | --- | --- | --- | --- | --- | --- | --- | --- | --- | --- | --- |
|  | **Criteria 1** | **Criteria 2** | **Criteria 3** | **Criteria 4** |  | **Criteria 1** | **Criteria 1** | **Criteria 2** | **Criteria 3** |  |  |
| Albuquerque PL et al | * | N/A | 0 | N/A | * | 0 | * | N/A | N/A | * | Poor |
| Arfaoui et al | * | N/A | * | N/A | ** | ** | * | N/A | N/A | * | Good |
| Bhargava S et al | * | N/A | 0 | N/A | * | 0 | * | N/A | N/A | * | Poor |
| Buchanan et al | * | N/A | * | N/A | ** | 0 | * | N/A | N/A | * | Poor |
| Ceesay B et al | * | N/A | * | N/A | ** | 0 | * | N/A | N/A | * | Poor |
| Ceron K et al | * | N/A | * | N/A | ** | 0 | * | N/A | N/A | * | Poor |
| Chafiq F et al | * | N/A | * | N/A | ** | 0 | * | N/A | N/A | * | Poor |
| Chen CK et al | * | N/A | * | N/A | ** | 0 | * | N/A | N/A | * | Poor |
| Chippaux JP | * | N/A | * | N/A | ** | 0 | * | N/A | N/A | * | Poor |
| Costa M et al | * | N/A | * | N/A | ** | ** | * | N/A | N/A | * | Good |
| Curic I et al | * | N/A | * | N/A | ** | * | * | N/A | N/A | * | Fair |
| Currie BJ et al | * | N/A | * | N/A | ** | 0 | * | N/A | N/A | * | Poor |
| Oliveira HFA et al | * | N/A | * | N/A | ** | 0 | * | N/A | N/A | * | Poor |
| Dehghani R et al | * | N/A | * | N/A | ** | 0 | * | N/A | N/A | * | Poor |
| Ebrahimi V et al | * | N/A | * | N/A | ** | * | * | N/A | N/A | * | Fair |
| Gampini S et al | * | N/A | * | N/A | ** | 0 | * | N/A | N/A | * | Poor |
| Gonzalez-Andrade F and Chippaux JP | * | N/A | * | N/A | ** | 0 | * | N/A | N/A | * | Poor |
| Gupt A et al | * | N/A | * | N/A | ** | 0 | * | N/A | N/A | * | Poor |
| Iliev YT et al | * | N/A | * | N/A | ** | 0 | * | N/A | N/A | * | Poor |
| Johnston CI et al | * | N/A | * | N/A | ** | 0 | * | N/A | N/A | * | Poor |
| Leite Rde S et al | * | N/A | * | N/A | ** | 0 | * | N/A | N/A | * | Poor |
| Machado C et al | * | N/A | * | N/A | ** | 0 | * | N/A | N/A | * | Poor |
| Pandey DP et al | * | N/A | * | N/A | ** | 0 | * | N/A | N/A | * | Poor |
| Patino RSP et al | * | N/A | * | N/A | ** | * | * | N/A | N/A | * | Fair |
| Tavares AV et al | * | N/A | * | N/A | ** | 0 | * | N/A | N/A | * | Poor |
| Rai A et al | * | N/A | * | N/A | ** | ** | * | N/A | N/A | * | Good |
| Sarkhel S et al | * | N/A | * | N/A | ** | 0 | * | N/A | N/A | * | Poor |
| Hattimy et al | * | N/A | * | N/A | ** | 0 | * | N/A | N/A | * | Poor |
| H. Khalid and R. S. Azrag | * | N/A | * | N/A | ** | 0 | * | N/A | N/A | * | Poor |
| Wood et al | * | N/A | * | N/A | ** | 0 | * | N/A | N/A | * | Poor |
| Farooq et al | * | N/A | * | N/A | ** | 0 | * | N/A | N/A | * | Poor |
| Hansson et al | * | N/A | * | N/A | ** | * | * | N/A | N/A | * | Fair |
| Cesaretli et al | * | N/A | * | N/A | ** | 0 | * | N/A | N/A | * | Poor |
| Roriz et al | * | N/A | * | N/A | ** | 0 | * | N/A | N/A | * | Poor |
| Lucsic B et al | * | N/A | * | N/A | ** | 0 | * | N/A | N/A | * | Poor |
| Dehghani et al | * | N/A | * | N/A | ** | 0 | * | N/A | N/A | * | Poor |
| Karki et al | * | N/A | 0 | N/A | * | 0 | * | N/A | N/A | * | Poor |

S3 (B): Assessment of studies (Cohort) using the Newcastle-Ottawa Scale (NOS) tool; *(Out of 9) Good quality: 3 or 4 stars in selection domain AND 1 or 2 stars in comparability domain AND 2 or 3 stars in outcome/exposure domain Fair quality: 2 stars in selection domain AND 1 or 2 stars in comparability domain AND 2 or 3 stars in outcome/exposure domain Poor quality: 0 or 1 star in selection domain OR 0 stars in comparability domain OR 0 or 1 stars in outcome/exposure domain

| **Study author** | **Selection (maximum 5)** | | | | **Comparability (maximum 1)** | **Outcome (maximum 3)** | | **Total score (out of 9)** | **Quality** |
| --- | --- | --- | --- | --- | --- | --- | --- | --- | --- |
|  | **Criteria 1** | **Criteria 2** | **Criteria 3** | **Criteria 4** | **Criteria 5** | **Criteria 6** | **Criteria 7** |  |  |
| Alcoba G et al | 1 | 1 | 0 | 1 | 1 | 1 | 1 | 6 | Fair |
| Alcoba G et al | 1 | 1 | 1 | 1 | 1 | 1 | 1 | 7 | Good |
| Gajbhiye R et al | 1 | 0 | 0 | 1 | 0 | 2 | 0 | 4 | Fair |
| Mahmood MA et al | 1 | 1 | 0 | 1 | 0 | 1 | 0 | 4 | Fair |
| Rahman R et al | 1 | 1 | 1 | 1 | 1 | 1 | 1 | 7 | Good |
| Senek MZF et al | 1 | 1 | 0 | 1 | 1 | 2 | 1 | 7 | Good |
| Vongphoumy I et al | 1 | 1 | 1 | 1 | 0 | 2 | 1 | 7 | Good |
| Hossain J et al | 1 | 0 | 1 | 1 | 0 | 1 | 1 | 5 | Fair |
| Parajuli et al | 1 | 0 | 1 | 1 | 0 | 0 | 1 | 4 | Fair |
| Majumder et al | 1 | 1 | 0 | 1 | 0 | 2 | 1 | 6 | Fair |
| Ediriweera et al | 1 | 1 | 1 | 1 | 1 | 1 | 1 | 7 | Good |

S3 (C): Assessment of studies (Cross-sectional) using the Newcastle-Ottawa Scale (NOS) tool; Grading: good: 7-9, fair: 4-6, poor: 0-3
